# Individual HLA heterogeneity and its implications for cellular immune evasion in cancer and beyond

**DOI:** 10.3389/fimmu.2022.944872

**Published:** 2022-09-05

**Authors:** Simona Pagliuca, Carmelo Gurnari, Marie Thérèse Rubio, Valeria Visconte, Tobias L. Lenz

**Affiliations:** ^1^ Translational Hematology and Oncology Research Department, Cleveland Clinic, Cleveland, OH, United States; ^2^ Service d’hématologie Clinique, Hôpital Brabois, CHRU Nancy and CNRS UMR 7365 IMoPa, Biopole de l’Université de Loarraine, Vandoeuvre les Nancy, France; ^3^ Department of Biomedicine and Prevention, University of Rome Tor Vergata, Rome, Italy; ^4^ Research Unit for Evolutionary Immunogenomics, Department of Biology, University of Hamburg, Hamburg, Germany

**Keywords:** HLA, immunopeptidome, HLA evolutionary divergence, tumor surveillance, immune escape

## Abstract

Structural and functional variability of human leukocyte antigen (HLA) is the foundation for competent adaptive immune responses against pathogen and tumor antigens as it assures the breadth of the presented immune-peptidome, theoretically sustaining an efficient and diverse T cell response. This variability is presumably the result of the continuous selection by pathogens, which over the course of evolution shaped the adaptive immune system favoring the assortment of a hyper-polymorphic HLA system able to elaborate efficient immune responses. Any genetic alteration affecting this diversity may lead to pathological processes, perturbing antigen presentation capabilities, T-cell reactivity and, to some extent, natural killer cell functionality. A highly variable germline HLA genotype can convey immunogenetic protection against infections, be associated with tumor surveillance or influence response to anti-neoplastic treatments. In contrast, somatic aberrations of HLA loci, rearranging the original germline configuration, theoretically decreasing its variability, can facilitate mechanisms of immune escape that promote tumor growth and immune resistance.

The purpose of the present review is to provide a unified and up-to-date overview of the pathophysiological consequences related to the perturbations of the genomic heterogeneity of HLA complexes and their impact on human diseases, with a special focus on cancer.

## Highlights

• Variability of human leukocyte antigen (HLA) genes underlies differences in presentation of antigenic peptides, influencing the risk for autoimmune diseases, infection and cancer
• The high heterogeneity of HLA molecules and consequently high HLA evolutionary divergence (HED) within an individual ensures the presentation of a broad immune peptidome
• Immune surveillance targets neoantigens in tumor cells and thus selects for somatic alterations of HLA and the antigen presentation machinery
• Somatic alterations of HLA heterogeneity can impair cancer or autoimmune target recognition by T-cell responses providing an immune escape environment
• Characterizing germline and somatic HLA heterogeneity can facilitate therapy decisions for cancer patients

## 1 Discovery of the major histocompatibility complex: The evolution of ideas

Antigen presentation *via* major histocompatibility complex (MHC) glycoproteins to T and, in part, natural killer (NK) cells, constitutes the basis for the processes of specific immune surveillance and central tolerance in most mammalian species (1–3).

**Figure 1:**
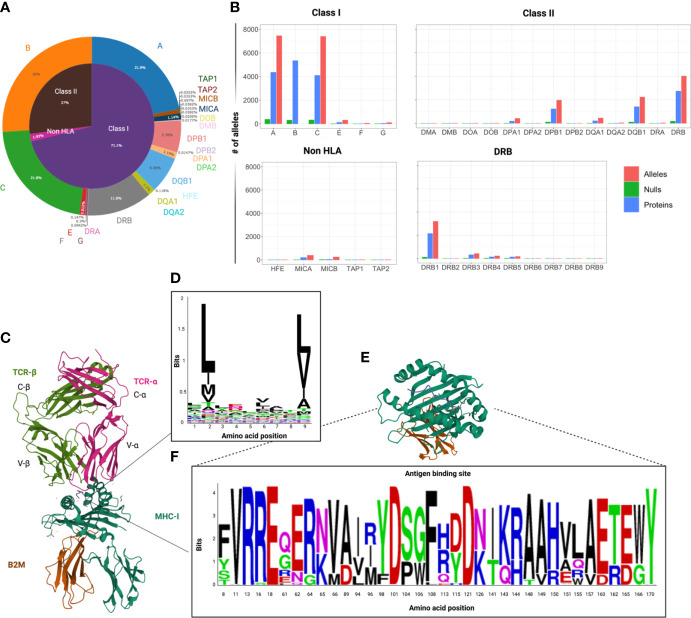
Variability of HLA locus. **(A)** Circle graph capturing the distribution of the number of HLA class I, class II and non-HLA alleles known to date within the human MHC region. Data extracted from the IPD-IMGT/HLA database v.3.48, downloaded in July 2022. **(B)** Bar graphs depicting the distribution of the number of alleles, proteins and null alleles (characterized by the presence of truncating mutations), per locus among classical and non-classical HLA genes. Of note is that HLA-DRB1 is responsible for the greatest variability within the copy number-variable DRB locus. **(C)** 3D crystallographic structure of TCR αβ chains interacting with an MHC class I molecule (allele A*02:01 and β2 microglobulin – β2M). These structures were extracted from the Protein databank (PDB) website and reoriented in order to show MHC-TCR synapsis (ref PDB:4MNQ) (10). **(D)** Representation of the HLA binding motifs of selected epitopes from a HLA-A2 related dataset, extracted from the IEDB (11) an visualized with Weblogo (https://weblogo.berkeley.edu ) **(E)** Crystallographic structure of the antigen binding domains of HLA-A*01:01 with some variable residues highlighted. **(F)** WebLogo visualization of the variable amino acids within the peptide binding domains (corresponding to exon 2 and 3) of the HLA-A alleles in a representative population of healthy controls (N=960). (12) The height of each letter corresponds to the relative frequency of each amino acid in the population. This graphic represents an attempt to visualize the variability of the antigen binding domain of HLA-A alleles, but does not capture the whole spectrum of heterogeneity, since it represents only a limited population.

The historical background leading to the discovery of the MHC complex is characterized by a number of landmark studies carried out during the first half of the XX^th^ Century on the role of the immune system in rejection or retention of tissue allografts, first in animal models and later in humans. Pioneers of these researches were (among others) Peter Medawar, Peter Gorer, George Snell, Jean Dausset, Jon van Rood, Rose Payne, who contributed to the understanding of fundamental concepts of transplant immunology (4–9) In 1964 the International Histocompatibility Workshop (IHW) was established to enable the progressive characterization of the MHC gene cluster and the identification of serologically defined alleles, contributing to portraying the medical and evolutionary significance of HLA polymorphisms, their relationship with human diseases and their importance in solid organ and allogeneic hematopoietic cell transplantation (HCT). In the following decades, the gradual advancement of molecular genomics allowed the identification of an incredibly high level of polymorphisms responsible for the exceptional variability of this gene cluster, which to date encompasses more than 33,000 different alleles among classical and non-classical HLA genes (IPD-IMGT/HLA database, v3.48, July 2022, **Figure 1**) (13). This exceptional variability has also triggered increasing MHC research in a wide range of non-model vertebrate species, which has advanced our understanding of immune system evolution in natural populations (14, 15). Overall, the intensive research of the last decades has contributed to define how genetic variations within the MHC region may determine predisposition to diseases, and the study of the functional consequences of this immunogenetic diversity is now a paradigm for human and evolutionary genomics (16, 17).

Since the discovery of MHC restriction, two major concepts have been invoked to explain the high MHC genetic variability and the related fitness advantage of diverse genotypes, namely the heterozygote advantage and the divergent allele advantage hypotheses (18–21). Both theories posit that presenting more different peptides is advantageous, but the latter, which is an extension of the former, stems from the observation that the more different two homologous HLA molecules are, the higher is their joint capability of presenting a broader infectious or cancer peptidome (20, 21). This feature is particularly important for the elaboration of antigen-specific adaptive responses and finds implications in many aspects at the basis of immune competence and central tolerance.

## 2 Structure and evolution of the human MHC gene cluster

**Figure 2:**
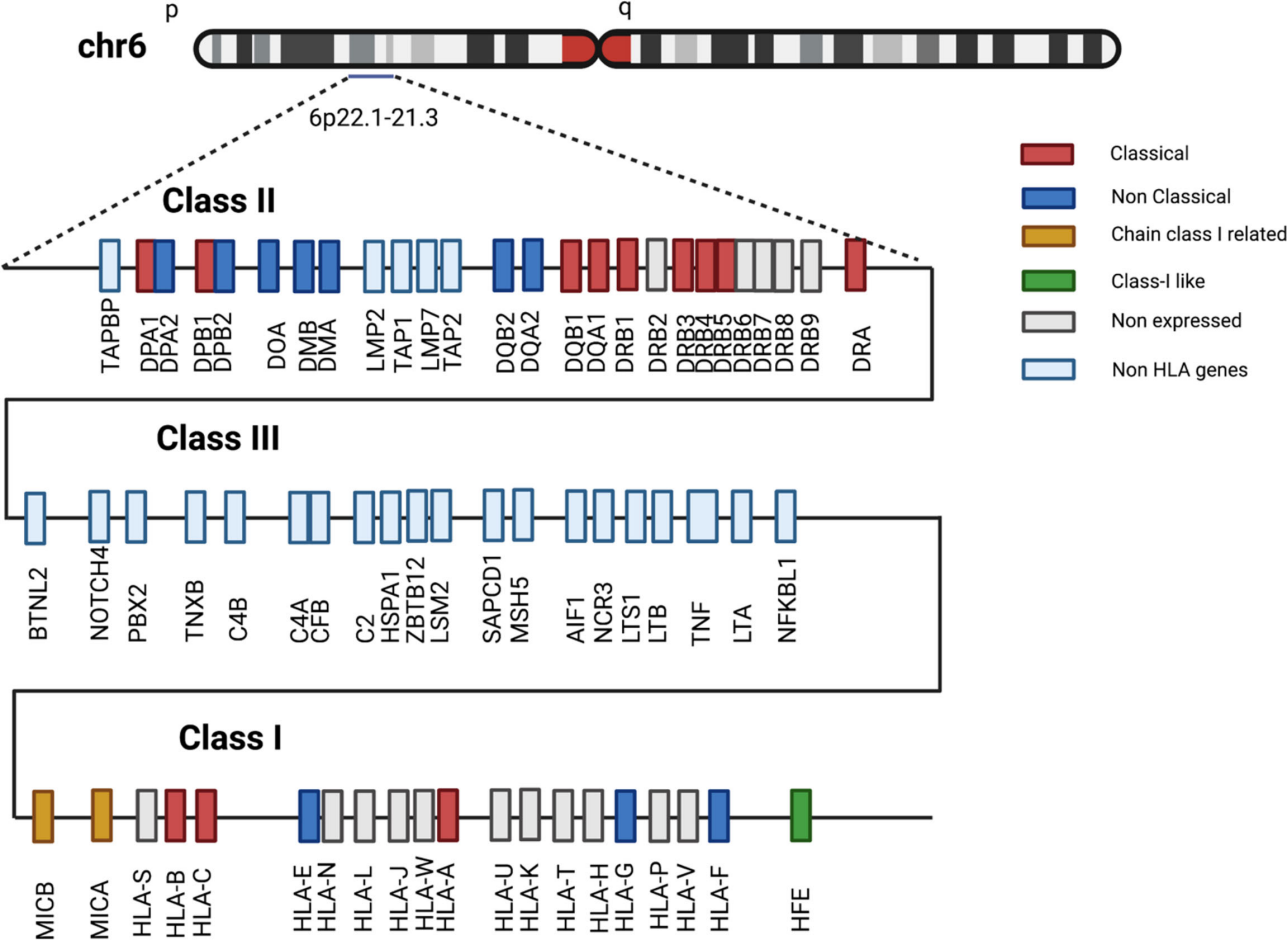
Schematic structure of the human MHC region. Selection of class I, II and III HLA and non-HLA genes located in 6p22.1-21.3 region. Gene-locus classification reported in legend follows IPD-IMGT/HLA database description. Distances among genes not to scale.

The MHC genomic cluster in humans includes more than 280 genes and encompasses a region of approximately 4 Mb, embedded on the short arm of chromosome 6 (6p21.3-22.1) (13). Three distinctive regions can be conventionally identified within this cluster, based on the topographical and functional homology of the genes involved: class I, II and III (**Figure 2**). The class I region contains classical (HLA-A, B, C) and non-classical (HLA-E, F, G) class I loci, and MHC class I chain-related genes (MICA, MICB, MICC, MICE, HFE) together with class I pseudogenes (H, J, K, L, N, P, S, T, U, V, W, Y), and non-HLA genes not related to immunity. The class II region comprises loci encoding α and β chains of HLA-DQ, -DR, and -DP molecules and other genes associated with antigen degradation, transportation and assembly (including the IFN-γ inducible immune proteasome components LMP2 and LMP7 and the transporters TAP1 and TAP2). An extended class II region incorporates a few genes with immune functions, including TAP Binding Protein (TAPBP or tapasin) and Retinoid X Receptor Beta (RXRB), associated with MHC-class I post-translational processing and transcriptional regulation, respectively. Finally, the class III region is densely populated by genes with innate and adaptive immune functions, including, among others, loci for the complement components C2 and C4A-B, tumor necrosis factor (TNF) and heat shock protein family A (HSPA1).

The study of patterns of inter-species conservation of these genomic regions uncovers interesting aspects associated with the evolution of the adaptive immune system. Some non-mammalian vertebrates, such as chickens, carry only a rudimental adaptive immune system in which only one MHC of each class is dominantly expressed, genes encoding proteins for antigen processing machinery (TAP and TAPBP) are very polymorphic and located next to class I MHC loci. This logistic genomic relationship suggests that the coevolution of these two clusters is of crucial importance for their function, likely defining their specificities for immune responses and resistance to pathogens (22, 23). Conversely, in humans and most mammalian species characterized by a fully developed adaptive immune system, specificities against pathogens are essentially based on the hyper-polymorphic variable regions within class I and II MHC loci, while genes encoding transporters and other members of the antigen presenting complex are characterized by a low (if not null) variability. These genes are also located far from the class I region, pointing towards reduced chances of co-evolution and diversification and underscoring the broader interaction of one or few genomic products with multiple class I alleles (22). Such differences in the genomic architecture of the MHC cluster across the phylogenetic tree represent a by-product of the evolutionary forces shaping vertebrate immune systems. Indeed, mechanisms of genome wide duplication, rearrangements, genetic drift and balancing selection are responsible for the complex hyper-polymorphic mammalian and human MHC systems, whose main feature is the capability to adapt to a wide range of pathogens and develop complex immune responses (23–25).

The evolutionary step allowing for the introduction of an antigen-based immune specificity is assumed to be the integration of RAG transposable elements in the jawed vertebrates’ genome, ancestral transposons at the basis of RAG1 and RAG2 recombinase activity governing the processes of V(D)J recombination, responsible for the diversity of antibody and T cell receptor repertoires (TCR) (26). This evolutionary event has facilitated an adaptive immune system that is based on highly diversified antigen-recognition elements.

Introducing this evolutionary perspective on antigen-specific immunity is of crucial importance for understanding the immunogenetic risk for certain infectious and immune-related diseases and may help in tracking patterns of MHC dysfunction at the basis of cancer evolution or susceptibility to complex diseases.

## 3 Variability of antigen presenting structures and divergent allele advantage model

MHC class I and class II molecules have the ability to present to immune effectors a broad assortment of protein-derived antigenic peptides on the surface of cells and their molecular and structural variability guarantees the breadth of this immune-peptidome. The wide range of HLA polymorphisms currently present in human populations has been selected by evolutionary forces *via* a process of balancing selection, favoring alleles or allelic combinations able to sustain the burden of diverse pathogens to which human populations have been exposed to throughout their history (21, 27–29) The extent of this genetic variation, mainly consisting in an increased rate of single nucleotide polymorphisms (SNPs), peaks within the antigen binding sites, resulting from natural selection favoring the diversification of peptide-binding capabilities (13). The basis for this pathogen-mediated selection is represented by the unique pathogen-specific antigen repertoires that hardly overlap among species, so that each pathogen species challenges the immune system in a different way (30).

Different not mutually exclusive mechanisms of pathogen-mediated balancing selection have been proposed over the last decades to explain the exceptional variability of the HLA genes (15, 31, 32). Among those, the heterozygote advantage hypothesis, first formulated by Doherty and Zinkernagel in 1975, assumes that individuals with an heterozygous HLA genotype are able to present a wider range of peptides and consequently to support immune responses against a larger range of pathogens, compared to homozygous genotypes (19). This assumption is further expanded by the divergent allele advantage model, stipulating that homologous HLA alleles with higher sequence divergence have the capability to bind more diverse and less overlapping peptide repertoires than less divergent genotypes (20, 21, 33). Such theories led to a paradigm shift for our understanding of antigen-specific immunocompetence: the genomic and consequently structural diversity of HLA molecules assures the elaboration of more diversified T-cell responses allowing efficacious anti-infectious and anti-tumor surveillance (21, 33–37).

## 4 HLA evolutionary divergence and its clinical implications in cancer

**Figure 3:**
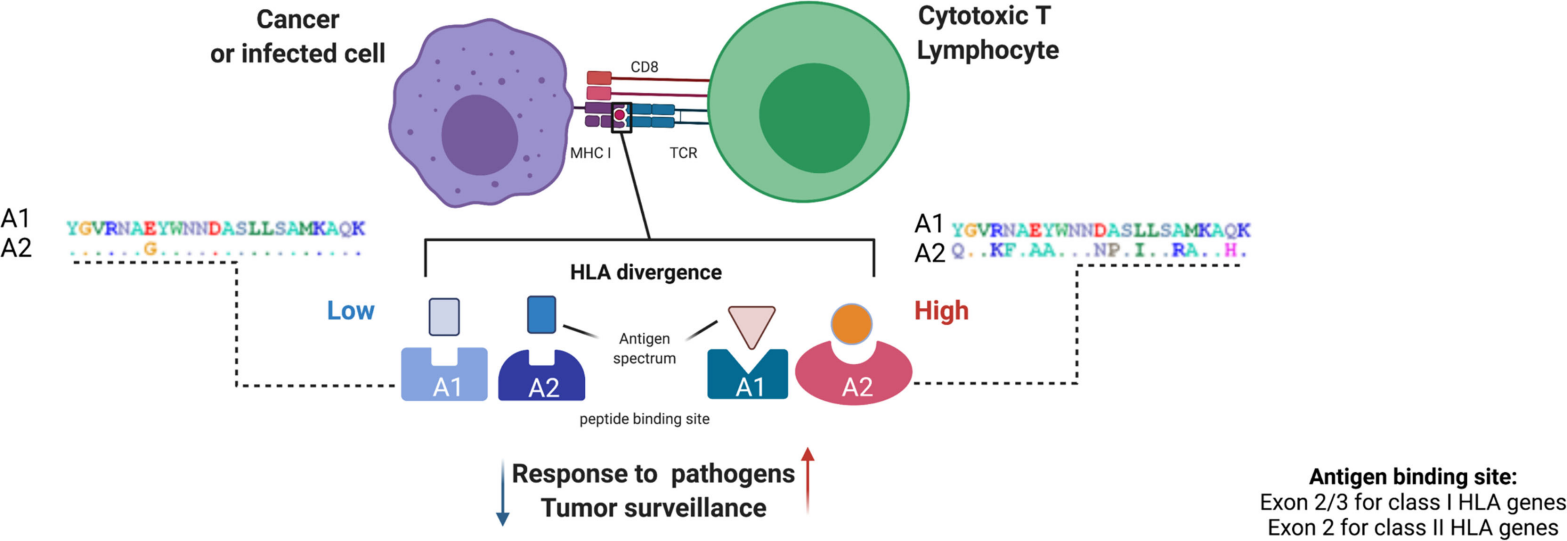
HLA evolutionary divergence calculation and pathophysiological implications. The HED score between two HLA alleles approximates the functional dissimilarity between the encoded molecule variants based on the Grantham distance between the amino acid sequences of their peptide binding domains. This computation is made using a dictionary including all the protein sequences of exons 2 and 3 for class I alleles and exon 2 for class II alleles, assembled from the IPD-IMGT/HLA database. (39) From a pathophysiological point of view, the divergence of HLA binding sites can influence patterns of antigen presentation in different processes, from response to infections to tumor surveillance, with a higher HLA divergence being associated with stronger immunocompetence and vice versa. At the right and the left as an example, A1 and A2 represent two alleles of the same locus. Each letter of A1 indicates an amino acid in the peptide binding site. In A2 letters designate variable amino acids (polymorphisms) and dots denote constant residuals. A1 and A2 on the left harbor a lower variability (low number of polymorphic residues) as compared to A1 and A2 on the right (higher number of polymorphic amino acids). Figure created with Biorender.

HLA evolutionary divergence (HED) (21, 37), is a recently implemented metric to quantitatively assess the functional difference between two HLA molecules, based on the variance in the corresponding amino acid sequence that encodes the peptide-binding groove of the two given HLA molecules. The HED metric is based on the Grantham distance score, which predicts the functional distance between two amino acids based on their physio-chemical properties, and which has been proposed for the estimation of damaging or deleterious amino acid substitutions (38). As higher Grantham scores reflect higher functional amino acid distances, higher HED values define functionally more divergent HLA sequences and *vice versa* (**Figure 3**).

This quantitative parameter has rapidly found applicability in many contexts due to its ability to capture the breadth of the immune peptidome presented by two different HLA alleles and, *per extenso*, by an individual with a given HLA genotype.

A recent landmark study in the field of immunotherapy shows that cancer patients homozygous for at least one HLA class I locus present a restricted and less diversified repertoire of tumor-derived neoantigens to CD8^+^ T-cells compared to fully heterozygous individuals, defining an immunogenetic configuration that may reduce the chances to respond to checkpoint inhibitors (40). This restriction of the neoantigenic pool may translate in a lower immunogenicity of cancer cells resulting in a reduced stimulation of anti-tumoral T cell effectors in class I homozygous patients as compared to fully heterozygous cases. Interestingly, somatic loss of heterozygosity within HLA class I loci, a phenomenon observed in many tumor tissues, may mimic this configuration, possibly representing an independent predictor for poor survival and poor response to immunotherapeutic agents (41). These findings underlie how selective pressures derived from the presentation of immunodominant peptides may be associated with certain HLA genotypic patterns (40). Later on, the application of HED metrics demonstrated how advanced-stage melanoma patients with more divergent HLA class I alleles could respond better to immunotherapy, regardless of tumor burden (37). Similar effects can be assumed more generally, i.e. independent of immunotherapy: compelling evidence shows that HLA class I genotypes may sculpt tumor genomes by presenting more or less immunogenic neopeptides, while frequent cancer-related oncogenic mutations have been associated with breach in immunopeptidome presentation, enabling the expansion of transformed cells characterized by impaired presentation capabilities (42, 43). Furthermore, the oncogenic mutational profile of a tumor may depend on the interaction between HLA genotype and specific cancer-derived neoantigens (44). In fact, HLA class I and class II genotypes have been shown to influence mutational events in cancers in a Darwinian fashion, being complementary to each other in establishing the patterns of immune escape from both CD8^+^ and CD4^+^ T-cell responses (44, 45). Hence, the mutational landscape of a transformed cell depends on the concertation among tumor genomic instability, neoantigen HLA binding predilection, and anti-tumor responses. Also, alternative and aberrant splicing has been shown as an important mediator of the shape of the neoantigen landscape, further affecting HLA-restricted immune surveillance (46). In this view, it is understandable how individual variation of HLA genotypes may contribute to shape the dynamics of anti-tumor adaptive immune response, restraining tumor oncogenic potential, and/or inducing mechanisms of immune escape.

## 5 Notes on peptide binding prediction

Thanks to the advances in computational immuno-oncology tools, the availability and the integration of HLA and genetic information may enhance the prognostication and the design of therapeutic algorithms for cancer patients. There are now pipelines able to predict the production of neopeptides, their affinity with HLA molecules and potential immunogenicity. These computational approaches are advancing the field of cancer immunotherapies and precision oncology (39, 47–51). Despite limits of early pipelines especially for class II-restricted epitopes, associated with poor specificity, and narrow sets of HLA alleles used as training frameworks, enhanced algorithms have been developed to accrue the accuracy of peptide characterization and immunogenicity quantification, including for class II-related antigens (49, 52–54). The precise characterization of HLA class I and II binding clefts plays a pivotal role in neoantigen prediction. In fact, key residues of the antigen binding site of HLA molecules may determine the binding motifs of the respective T cell epitopes. The study of the physiochemical composition of these motifs may be deployed to enhance binding prediction performance (55). In addition, characterization of complementarity determining 3 regions (CDR3) within the variable portion of TCR beta chains may be used to enhance reverse prediction of bound antigens (10, 56, 57). Theoretically one could predict the characteristics and the specificity of each epitope (i.e. autoimmune, cancer, pathogen-related), the related bound HLA alleles, or the function of the recognized T-cells (i.e. CD4 vs CD8) (58). Nonetheless because of the limited number of HLA alleles, disease and T-cell subtypes tested in experimental conditions, the identification of each motif group and, consequently, epitopes’ characterization and prediction to date remain challenging. As an example, we reported in **Figure 1** the representation of the HLA-A2 restricted binding motifs of selected nonameric peptides, extracted from The Immune Epitope Database (IEDB) (11).

## 6 Impact of HLA variability on NK reactivity and role of non-classical HLA peptide presentation

If HLA-antigen-TCR interactions restrict the specificity of adaptive immunity, HLA variability can also impact tumor surveillance *via* the innate strand of the immune system characterized by NK control. NK cells are capable of integrating and elaborating activating and inhibitory signals, thus modulating their reactivity against compromised cells according to the intensity of the feedback (59). Specifically, NK cells recognize self-peptides derived from classical and non-classical HLA, adhesion molecules and others *via* activating or inhibitory killer cell immunoglobulin-like receptors (KIRs) (60). Allelic heterogeneity directly influencing the surface density and binding affinities, weights the global avidity of KIR-HLA interactions (61). NK cells are also able to recognize patterns or molecular configurations characterizing transformed cells, (for instance downmodulation of HLA class I molecules – “missing self”), thereby sparing healthy tissues (59, 62, 63). These aspects are particularly important in NK-based immune surveillance and may shape the degree of anti-tumor control defining an “immunogenetic” predisposition to develop solid tumors, respond to immunotherapy, or induce post-HCT alloreactivity (64–68).

Non-classical class I HLA molecules (in particular HLA-E, G and F) have shown an important role in both immune surveillance and tolerance, essentially displaying regulatory functions towards NK and T cell activation. Although similar in structure to classical class I molecules, non-classical HLA class I alleles are oligomorphic and often exhibit a limited tissue distribution, modulated in inflammatory conditions. The decreased variability accounts for a limited immune peptidome repertoire and specific immunoregulatory functions. In physiological conditions, HLA-E binds self-peptides generated from the peptide leader sequence of class I molecules (HLA-A, HLA-B, HLA-C and HLA-G), participating in immune tolerance (69, 70). Other non-classical HLA molecules display instead antigen-presenting features, interacting with specific α/β TCRs and intervening in adaptive immunity (69). HLA-G has been linked to immunosuppressive phenomena by inhibiting NK and T-cytotoxic activation and chemotaxis (71, 72). Variation at the HLA-G locus has been demonstrated as affecting its mRNA translation and stability, impacting on tumor susceptibility or treatment outcomes (73–75).

## 7 Somatic dysfunction of HLA diversity: HLA loss and beyond

Downregulation of the antigen presentation machinery represents a paradigm of evasion from anti-tumor immune surveillance.

Large genomic aberrations, fine somatic mutations, epigenetic silencing and transcriptional modifications involving directly the HLA region or genes encompassing antigen degradation, transportation and processing have been invoked across large cohorts of solid tumors and hematological disorders, all as a result of a common lynchpin: the selective pressure induced by the antigen-specific adaptive immune system.

**Figure 4:**
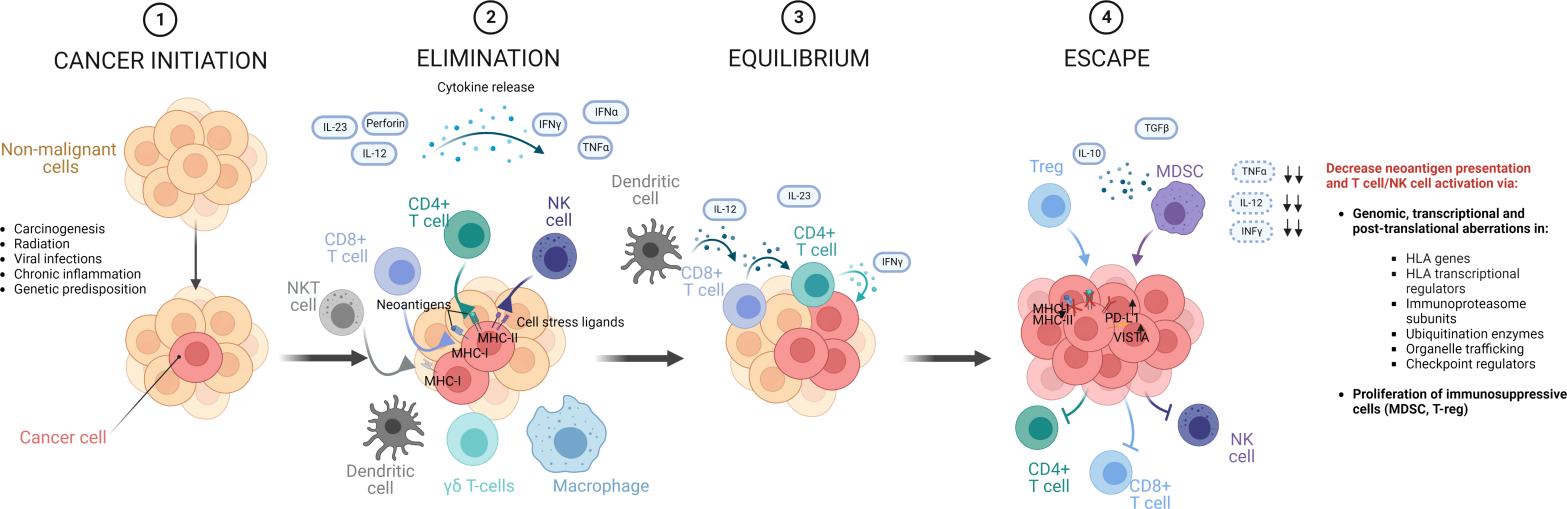
Phases of cancer immunoediting. In the natural history of a tumor, aberrant cells that fail intrinsic mechanisms of control and tumor suppression, are exposed to an external control operated by innate and adaptive immunities, which initially contain cancer progression, contributing to sculpt its genomics, by eliminating cells with higher immunogenic potential. This immunological mechanism starts a process called immunoediting, in which cancer cells are exposed to T-cell and NK selective pressures. This phase of elimination is in general followed by an equilibrium phase, where all the mechanisms of tumor-surveillance are operative and constrain tumor cells, reaching a balance. In this context, rare cells can develop immune resistance, ultimately reducing the presentation of immunogenic neoantigens and T cell activation and entering into an escape phase in which tumor progression takes over, because not restricted anymore by the immune system. Figure created with BioRender.com

To better understand the complex mechanisms underpinning immune escape, it is important to consider the fascinating concept of *immunoediting* deriving from the study of immune responses in tumor physiopathology. Cancer immunoediting is a complex network of events that characterizes the double capability of tumor immune surveillance to both coerce and endorse tumor growth, by inducing evolutionary pressures culminating in specific patterns of clonal selection (76–78). Three phases can be identified in this pathophysiological phenomenon after cancer initiation: elimination, equilibrium and escape (**Figure 4**) (76).

In a first phase of the development of a cancer, both innate and adaptive immune system can identify and destroy cells undergoing processes of malignant transformation that evade intrinsic cellular mechanisms of control (77). This initial phase of tumor surveillance contributes to sculpt the tumor genomic landscape, eliminating clones with more immunogenic mutational products (e.g., *via* HLA-presentation of neoantigens with high capabilities to elicit T-cell responses). Such a process may last for a long time (equilibrium phase) during which tumor surveillance opposes cancer progression. However, the constant pressure operated by the immune surveillance on the instable cancer genome may culminate in genomic or transcriptional aberrations in HLA, antigen presenting and processing machinery- or checkpoint- related genes (e.g., HLA mutations, PDL1 upregulation), allowing tumor cells to evade immune surveillance (76). Such immune-edited tumors can then enter into the final escape phase, characterized by an uncontrolled proliferation, after destabilization and exhaustion of immune surveillance mechanisms (76). The consequences of this multiphase process become clear when characterizing responses to immunotherapy. Failure or resistance to immunomodulatory strategies in cancer treatment can in fact derive from the development of such mechanisms of immune evasion, and their molecular characterization is suggested to clinically predict the response to such targeted therapies (76, 79).

Genomic aberrations involving the HLA region, such as the loss of an entire haplotype or an allele, have been described in different solid cancers and hematological malignancies and may occur through mechanisms of deletions or copy-neutral loss of heterozygosity (CN-LOH) of chromosome 6p21 (80–85) Recently, somatic mutations in HLA class I genes have been identified as important contributors in the landscape of cancer immune-evasion, especially for tumors characterized by upregulation of T-cell cytotoxic activity signatures and high immunoediting potential (86).

Several other mechanisms underlying down-regulation or loss of HLA class I in human tumors have been described, resulting in many different phenotypes (87). Total loss of HLA class I presentation may also be due to mutations in β2M or in other genes involved in the antigen-presenting machinery, such as immunoproteasome subunits LMP2 and LMP7 or the transporters TAP-1 and TAP-2 (88, 89).

A paradigmatic example of how somatic aberrations in the HLA region can lead to immune escape *via* abolition of the presentation of immunogenic peptides is the CN-LOH of the non-matched HLA allele in allogeneic HCT. Such a complex genomic phenomenon, which occurs in the setting of mismatched HCT transplants, underlies the duplication of the matched allele replacing the mismatched gene in recipient-derived hematopoiesis, as mechanism of leukemic relapse (90, 91). In this context, the elimination of the incompatible HLA alleles by malignant cells, would restrain the presentation of immunogenic alloreactive peptides, without decreasing the overall level of expression of HLA class I molecules. As a result, NK cell recognition, which is principally activated in response to the absence or downregulation of self-HLA class I molecules, would be disabled, as shown with functional essays in the setting of acute myeloid leukemia relapse after haploidentical transplantation (91). Therefore, the anti-tumoral effect of the allogeneic HCT (*graft versus leukemia effect*) is circumvented, allowing disease relapse.

## 8 Immunoediting forces shaping clonal evolution: A common lynchpin in cancer and autoimmunity?

As mentioned above, the HLA-restricted antigen-specific immune response exerts a mechanistic pressure against unknown and non-self-peptides and, by this virtue, cancer cells harboring mutated, strong immunogenic proteins are eliminated. This process underscores an important pathophysiological aspect. Tumors with higher levels of immune infiltration may have (at least at an initial stage of immunoediting) a lower mutational burden, in line with the observed better outcomes of patients whose cancers are characterized by stronger immune signatures (92–94). The key for tumor cells to achieve a clonal advantage would thus be to lose the capability for presenting immunogenic peptides without losing their fitting advantage or proliferation propensity. As a consequence of such impaired immune restriction, tumors adapting this way to evade immune surveillance may develop a different molecular landscape, with higher mutational burden and genomic instability, compared to those that are still evolving under a competent antigen presenting machinery (95, 96).

In tumor biology, the down-modulation of HLA-restricted immunity becomes an example of evolutionary adaptation established by mutated cells undergoing immune selection. In this context, one of the multiple outstanding issues is how to treat cancer patients who present with “the stigma” of immune escape, i.e. whose tumor has developed one or more immune evasion phenotypes. For instance, in case of disease recurrence after allo-HCT, a lower response rate to donor lymphocyte infusion and other immunomodulatory strategies has been shown in case of loss of the mismatched haplotype, with a slight improvement in survival when patients receive a second transplant from a different donor, thereby re-establishing the HLA heterogeneity balance (91, 97). Also, in pan-cancer studies, loss of HLA class I has been described as a negative predictor of survival and response to immune checkpoint inhibitor treatment (40, 41) pinpointing an important pathophysiological aspect: highly immune-edited tumors exhibiting features of immune escape are less likely to respond to pharmacological T-cell activation. It is also noteworthy to recognize the complexity of the association between immune escape and tumor mutational burden (TMB): a recent pan-cancer study suggested that the prevalence of HLA class I loss may follow a “Goldilocks” pattern. Indeed, tumors with the highest (i.e., cutaneous melanoma) and lowest TMB (i.e., neuroendocrine tumors) had lower incidence of HLA losses as compared to malignancies with an intermediate TMB, underscoring the non-linearity of this association and the possibility of additional patterns of evasion from immune recognition in highly instable tumors (41).

All these observations are in line with the inconsistent immunotherapy efficacy across cancer cohorts and with the unmet clinical need for providing non-HLA restricted immunomodulatory strategies overcoming the above-mentioned issues. An important example of non-HLA restricted immunotherapy is represented by genetically engineered T cells (TCRs and chimeric antigen receptor -CAR- T cells), or bispecific T cell engager therapies (BiTE) whose specificity is designed to be directed against precise tumor neoantigens bypassing MHC restricted epitope presentation by means of modified T cell surface molecules able to target specific overexpressed cancer antigens (i.e. CD19 in lymphoproliferative disorders) (98, 99). Nonetheless it is noteworthy to highlight that similarly to the pression induced by the HLA-restricted immune selection, conducting to somatic rearrangement of HLA region and loss of immune-dominant antigen presentation, one of the major mechanisms of treatment resistance is the down-modulation of the targeted molecule. This process may represent another operative mechanism of immune antigen escape, associated with the pressure exerted by CAR-T cells or BiTEs on their target (98, 100).

As said, NK cells exert their anti-tumor activity in a way mostly independent of the presentation of specific neoantigens, being still able to discriminate between healthy and transformed cells. Due to this ability, there is a rapidly emergent interest in developing engineered CAR-NK cells as a strategy of cancer cellular-therapy. The short life, the safety profile particularly with regards to the absence of major cytokine release, and the innate cytolytic activity against a great variety of cancers render CAR-NK-based platforms particularly attractive for implementation in different clinical settings of onco-hematology (101–103).

Clonal evolution can theoretically occur in all types of cancer where the immune system exerts an initial control on the neoplastic cells. One could speculate that similar mechanisms of immune escape may be present at a certain level also in some autoimmune disorders. A prototypic example in this direction is represented by idiopathic aplastic anemia (IAA), an immune mediated bone marrow failure disease characterized by T cell mediated destruction of hematopoietic stem cells, translating in failure of hematopoiesis (12, 77, 80, 81, 89, 104, 105). Here, genomic loss of HLA molecules has been shown as a frequent mode of immune escape of target cells from autoimmune attack, theoretically lowering the presentation of immunogenic peptides below the activation threshold of T-cell effectors. Autoimmunity is not generally associated with high mutational rates in targeted tissues and the development of neoantigens, and besides IAA, the possibility of immune evasion due to immune pressure has not been raised in other autoimmune diseases. However, certain aspects of the same immunoediting scenario seen in tumor biology could also play a role in HLA-mediated autoimmunity, which is triggered by erroneous immune activation of adaptive responses against self-peptides, a process quite similar to the HLA-mediated targeting of neoantigens on tumor cells. While the HLA loss observed in IAA is an exemplificative epiphenomenon of such processes, one could speculate that this mechanism can be operative in other autoimmune contexts within the tissues selectively attacked by the immune system *via* HLA-restricted pathways, as a mechanism of “adaptive” rescue. Such clonal selection obviously requires some level of cell division, and can thus only operate in tissues with regenerative activity, but it might be worthwhile to explore this in appropriate autoimmune settings beyond IAA.

## 9 Conclusive remarks

Molecular dysfunction of individual HLA heterogeneity constitutes a common mechanism at the basis of a number of pathological processes encompassing infectious, neoplastic and possibly autoimmune pathophysiology. On one hand, the assessment of germline HLA genomic and structural diversity may have important prognostic implications as in the case of risk profiles associated with response to immunotherapy, or the propensity to develop certain autoimmune disorders. On the other hand, somatic loss of heterozygosity or reduced presentation of immunogenic peptides may configure patterns of immune escape in a variety of disorders of both neoplastic and non-malignant nature. Given the importance in human diseases, the analysis of HLA genotypic configurations and the relative somatic dysfunction should be integrated in clinical practice, especially in the immunology and onco-hematology fields, where their assessment may help in refining patients’ prognosis and the suitability for immunomodulatory agents’ interventions.

## Author contributions

SP reviewed available literature and wrote the manuscript. CG, MR, VV and TL provided critical discussion and revised and edit the manuscript draft. All the authors conceptualized the study and agreed to the published version of the manuscript.

## Funding

We acknowledge the following sources of funding: AA & MDSIF, VeloSano Pilot Award, and Vera and Joseph Dresner Foundation–MDS (to VV), Italian Society of Hematology, Italian Society of Experimental Hematology, Fondation ARC pour la recherche sur le cancer (to SP), American-Italian cancer foundation (to CG), Deutsche Forschungsgemeinschaft (DFG, German Research Foundation) – 437857095 (to TL).

## Acknowledgments

We thank all the reviewers who contributed with constructive comments to improve this work.

## Conflict of interest

TL is co-inventor on a patent application for using HED as a prognostic marker for immunotherapy success.

The remaining authors declare that the research was conducted in the absence of any commercial or financial relationships that could be constructed as a potential conflict of interest.

## Publisher’s note

All claims expressed in this article are solely those of the authors and do not necessarily represent those of their affiliated organizations, or those of the publisher, the editors and the reviewers. Any product that may be evaluated in this article, or claim that may be made by its manufacturer, is not guaranteed or endorsed by the publisher.
